# Effects of an Online Solution-Focused Psychoeducation Programme on Children’s Emotional Resilience and Problem-Solving Skills

**DOI:** 10.3389/fpsyg.2022.870464

**Published:** 2022-07-19

**Authors:** Melih Burak Özdemir, Ayşe Bengisoy

**Affiliations:** ^1^Department of Guidance and Psychological Counseling, Institute of Graduate Studies and Research, European University of Lefke, Mersin, Turkey; ^2^Department of Psychological Counseling and Guidance, Faculty of Education, European University of Lefke, Mersin, Turkey

**Keywords:** COVID-19, solution-focused, psychoeducation, online, children

## Abstract

This study investigates the effects of a solution-oriented and approach-based psychoeducation programme, initiated in December 2019 and conducted online during the worldwide COVID-19 pandemic, on children’s emotional resilience and problem-solving skills. In the world that was closed with the pandemic, children were kept away from the social support system of teachers and friends. Pandemic isolated the children. The negative experiences associated with COVID-19 have placed both traditional approaches and important online learning and support applications on the agenda, since both can help to solve the problems we face. Educators and experts have provided psychological support services, questioning the methods used to reach people during the pandemic and rediscovering alternative ways of connecting with individuals through online media. The online framework of this study emerged in response to the needs caused by the pandemic. The study is designed using a real experimental pattern and is based on a pretest-posttest, experimental/control-group model. The Social Support Evaluation Scale for Children and Teenagers was used to select the participants. In total, 18 participants were equally assigned to research groups (experiment *n* = 9, control *n* = 9). The psychoeducation programme consisted of six sessions, each lasting 50–60 min on average. Research carried out online during the pandemic shows that this psychoeducation programme is effective in strengthening students’ problem-solving and emotional resilience skills. These findings are discussed within the framework of the literature, followed by recommendations.

## Introduction

The COVID-19 pandemic began in late 2019 and soon became a worldwide hurricane, negatively impacting society, culture, politics, economics, and aspects of life that cannot yet be predicted. There is no doubt that the education sector has been in the middle of this hurricane. The pandemic has significantly changed our perspective on education (Bozkurt and Sharma, 2020). The fact that formal educational practices could not be maintained on a global scale led to the addition of open and distance-education practices as basic approaches to learning. Globally, the pandemic closed down face-to-face education on a global scale, causing millions of individuals to learn through open and distance education (Can, 2020). Faced with the closure of schools in every context, governments, teachers, students, caregivers, and parents found it extremely challenging to ensure continuity of education (Chang and Satako, 2020). Face-to-face training, which was suspended in the Turkish Republic of Northern Cyprus (TRNC) on 11 March 2020 due to the pandemic, resumed in September 2020. The schools, which closed again on 31 December 2020, reopened in September 2021.

The pandemic has made it necessary to try new modalities in many sectors. Art centres, sports fields, educational institutions, and shopping centres have been closed to prevent people from gathering; working from home was introduced to prevent people from gathering in workplaces. This has increased the need for online systems, while different ways of doing business have introduced new developments (Bozkurt et al., 2020). During this process, school counsellors have worked online, identifying student needs and problems, accessing student socio-demographic data, collecting exam and school data, and organising school-activity data (Supriyanto et al., 2020). The lack of opportunities for face-to-face psychological support during the COVID-19 pandemic has increased the need for online psychological counselling and guidance. As a consequence, negative attitudes about receiving and providing support online have begun to change (Yüksel Şahin, 2021).

Previous studies have shown that, although psychological counselling and other mental-health services existed online before the pandemic, they were less common than at present. They have become significantly more important during the pandemic (Ifdil et al., 2020). Most professionals who provide mental-health services incorporate technological methods into their professional practices. According to VandenBos and Williams (2000), 98% of the members of the American Psychological Association (APA) use technology when needed to provide therapeutic services. According to Zeren and Bulut (2018), the first person to offer innovative consultancy services by moving beyond traditional approaches was Freud, who experimented with a correspondence therapy process, using written communication in the case of “Little Hans.” After Freud, in 1968, telephone use increased, and the Massachusetts Hospital began to provide a very non-traditional psychiatric service, using the telephone.

Psychological support can be offered to individuals and groups. Group work can take the form of group counselling, group guidance, and psychoeducational activities (Zeren et al., 2020). This study is a group psychoeducation study conducted within the scope of group studies.

The COVID-19 pandemic severely limited face-to-face meetings and contact between individuals, causing an expansion of online mental healthcare services (Zhang and Ma, 2020). During the COVID-19 pandemic, as lives were restricted to the home, no one could predict how long the pandemic would last, when it would end, or the extent of its effects. Under these difficult conditions, the field of psychological counselling has been badly affected, as have many other fields. The extent to which technology has changed everyday life has paved the way for experts who provide psychological-support services to offer such services online. During the pandemic, new educational systems began to take shape on a world scale, as countries searched for ways to continue education without interruption. Technology reached the point where online processes began to replace face-to-face communication. Conditions required the closure of schools in the TRNC on 31 December 2020. The need to adapt to the rapid changes emerging from uncertainty made it necessary to conduct this experimental study online. The extensive use of online platforms during the pandemic was the main reason for conducting this study online.

The idea of problem-solving was first used by the American educator John Dewey in the field of education. Prawat (2000) summarised Dewey’s philosophy of education as follows: It is very important for students to gain problem-solving skills, in terms of fulfilling both individual and social functions of education, so that they can overcome all kinds of problems they encounter with. According to Ornsteın and Lasley (2000), students who are successful at problem-solving are in an effort to take action to reveal the problem and use their previous knowledge to solve it. From a developmental perspective, emotional resilience is defined as children’s ability to manage their stress when it comes to challenging life issues. In this respect, every stage of education should be supported with various activities and programmes that would help children’s problem-solving skills and emotional resilience. Through this, the development of children’s problem-solving skills is supported, and they gain the skills to recognise, understand, and manage their emotions.

A few important programmes on emotional indomitableness in the world are as follows: “You Can Do It,” which aims to maximise the emotional, social, and academic development of children and teaches children emotional indomitable skills. The programme was developed in Australia by Dr. Michael Bernard based on a cognitive behavioural approach. The programme has been implemented in nearly 1,500 schools in Australia, the United States, the United Kingdom, New Zealand, Romania, and Singapore for more than 10 years and has had successful results (Bernard, 2011). Another programme is “Friends for Life.” The programme was developed by Dr. Paula Barrett in Australia. This programme aims to overcome childhood depression and anxiety by strengthening emotional indomitableness; it is a school-based and socially oriented programme based on a cognitive behavioural approach. It has been practised for more than 15 years in many countries such as New Zealand, Germany, Saudi Arabia, China, the United States, the United Kingdom, Norway, Finland, the Netherlands, Portugal, and Canada (Stirling Council Educational Psychology Service, 2007).

Based on this information, the research question was as follows: “*Is a solution-focused psychoeducation programme conducted online during the COVID-19 pandemic effective in strengthening children’s problem-solving and emotional resilience skills?*”

No previous research has combined the problem-solving and emotional resilience skills of primary school students in a single study under pandemic conditions in the TRNC, within the framework of a psychoeducation programme based on an online solution-oriented approach. This study makes a valuable contribution to the literature and may have a guiding effect on future research.

## Methods

### Research Design

This experimental research study analyses the impact of a psychoeducation programme, based on a solution-oriented approach, on the problem-solving skills and emotional resilience of the fourth- and fifth-grade primary school students. The independent variable is a “six-session psychoeducation programme, based on the solution-oriented approach,” while the dependent variables are “problem-solving skills” and “the level of emotional resilience.”

The study incorporates an experimental design that includes a pretest-posttest and experimental and control groups. In this model, two groups are randomly assigned to the experimental and control groups. The study is designed using a real experimental pattern. Before the experimental study began, the entire psychoeducation programme was trialled as a pilot study using students outside the experimental and control groups; the programme was observed to function properly. It was then applied to the pilot and experimental groups online, since schools in the TRNC closed on 31 December 2020 due to the pandemic.

### Research Sample

The subjects were 110 fourth- and fifth-grade primary school students [51 (46.4%) were female subjects and 59 (53.6%) were male subjects], attending a public primary school in Kyrenia, TRNC, in 2020–2021. The school population was lower middle class; the experimental and control groups consisted of nine students each. The “Social Support Evaluation Scale” was applied to 110 students in the fourth and fifth grades according to the voluntary principle. As a result of the application, students whose social support score was below average were included in the process on a voluntary basis. Students who were below average were randomly assigned to the experimental and control groups.

### Data Collection Tools

The reliability of the scales used in the study (the Cronbach’s alpha internal consistency coefficients) was analysed using SPSS 24 software. It was initially examined because the population and the sample for which the scales were developed and applied differed from the study population and sample. The Cronbach’s alpha value was used to indicate the reliability and homogeneity of items in the scale (a scale with a high Cronbach’s alpha value is thought to consist of consistent items that measure similar characteristics). The Cronbach’s alpha coefficients were as follows for the scales used in this study:

The Social Support Evaluation Scale for Children and Teenagers: 0.87 (α = 0.87).
The Problem-Solving Inventory for Children: 0.72 (α = 0.72).
The Emotional Resilience Scale: 0.87 (α = 0.87).

### The Emotional Resilience Scale

As one of the three “resilience scales” (Prince Embury, 2007), the Emotional Resilience Scale (ERS) concentrates on the emotional dimension of resilience; the present version was translated and adapted for use in a Turkish setting (Kurtoǧlu, 2013). In terms of internal consistency, the Cronbach’s alpha coefficient was 0.83, and the test correlations for individual items ranged between 0.25 and 0.53. The scale consists of three factors. Kurtoǧlu (2013) performed the explanatory and confirmatory factor analysis to determine the validity of the scale. The results of both analyses confirmed the three-factor structure consisting of the sub-dimensions, namely, “Sensitivity,” “Healing-Improvement,” and “Deterioration.”

The original Emotional Resilience Scale (ERS) was a five-point Likert-type scale consisting of 20 items. In the present scale, which is tailored for children and teenagers between the ages of 8 and 14 years, a low score indicates a high level of emotional resilience and a high score indicates a low level of emotional resilience. Reverse coding was applied to all items to obtain the correct results and avoid misinterpretations in the study analyses. Therefore, students who obtained low scores were considered to have low emotional resilience, while those who obtained high scores had high levels of emotional resilience.

### The Problem-Solving Inventory for Children Attending Elementary School

This study used the Problem-Solving Inventory for Children Attending Elementary School (PSIC) developed by Serin et al. (2010) to measure the problem-solving skills of fourth- and fifth-grade students. The PSIC scores ranged from 24 to 120. The Cronbach’s alpha reliability coefficient for the whole scale was 0.80; the scale was a five-point Likert-type scale. The Cronbach’s alpha reliability coefficient for the whole scale was 0.83. A high total PSIC indicates that the subjects perceive themselves to be sufficiently capable of problem-solving. This scale, whose findings were valid and reliable, was the first assessment instrument developed in Turkey to determine how primary school students perceived their own problem-solving skills. The opinions of three expert lecturers, three classroom teachers, seven postgraduate students, and seven doctoral students were taken on the items in the scale to ensure the content validity of the scale and to check whether the subject that the scale wants to measure is suitable or not. In the light of the opinions and suggestions received, some items were removed from the scale, and necessary corrections were made to some of them. “Basic component analysis” was applied to test the construct validity of the scale.

### The Social Support Evaluation Scale for Children and Teenagers

The Social Support Evaluation Scale for Children and Teenagers (CT-SSES) was developed by Dubow and Ullman (1989) to evaluate children’s perceptions of the social support they receive from their families, friends, and teachers. It consists of items, based on the definition of social support, which assess the degree to which children consider themselves to be loved, cared for, valued, and accepted as individuals within their social network.

The Turkish form of the CT-SSES was tested by Gökler (2007), who assessed its psychometric properties for use with children and teenagers. The scale was judged to be suitable for children and teenagers aged between 9 and 17 years. The Cronbach’s alpha reliability coefficient for the whole scale was 0.93 (Gökler, 2007). Both the factor structure and the criterion validity of the scale were examined to determine its validity.

## Data Collection

Study data were collected in line with an experimental design implemented during the second semester of the 2020–2021 academic year. Prior to the experimental process, problem-solving and emotional resilience scales were applied to both groups included in the experimental design as a pretest. After applying the psychoeducation programme to the experimental group, the same data collection tools were again applied to the experimental and control groups as a posttest.

Throughout the study, students participated as volunteers. They were not pressured in any way; all participants were free to leave the study at any time. The school management and parents were informed before the research began.

### Process

Initially, a needs analysis was carried out to select participants for the study. Regarding the participants’ socio-economic level, the primary school in question catered to a lower middle-class population. Most of the participants were the children of parents who came to the island to work from countries such as Turkey, Russia, India, and Pakistan. The “Social Support Evaluation Scale” was applied on a voluntary basis to 110 fourth and fifth graders who had been referred to the counselling service, based on the observations of the school counsellor, classroom teacher, and branch teacher. The scale was implemented before schools’ closure due to the pandemic. The parents of students who received low social-support scores were then contacted by telephone. The process was explained to the parents, after which, students who wanted to participate voluntarily were provided with information on the programme content, process, volunteer nature of their participation, and confidentiality. Informed “parental permission” was obtained verbally from all parents due to the pandemic conditions. Members of the school administration were also informed of the process before the research began. For a study of this type, the ideal number of participants is 7–8, while 5–10 is considered acceptable. In this study, 9 students each were considered sufficient for the volunteer experimental and control groups. Two psychoeducation sessions per week were conducted online; group sessions were held between 15 May and 5 June 2021, with each session lasting approximately 50–60 min.

### Data Analysis

The data of the research were analysed using the SPSS 24 software package. To decide the parametric/non-parametric tests to be used in the study, whether or not the score distributions complied with normality and homogeneity assumptions was tested by calculating the pretest and posttest scores of the experimental and control groups. The independent sample *t*-test was applied to determine whether groups are equal in cases where the assumption of normality is achieved and variances are distributed homogeneously; the paired sample *t*-test was applied to analyse whether the final test scores differed. The importance level of the study was taken as 0.05.

### Ethical Issues

Ethics committee approval for the ethical appropriateness of this research was obtained from the European University of Lefke Ethics Committee with the decision number ÜEK/60/02/04/2021/01 dated 13 April 2021. Children’s volunteerism was taken into consideration during the study, and children were not forced to any application. The right to withdraw from the application was given to the children during the application period.

### The Programme Structure and Content

First, the hypothetical basis and the philosophical basis of the programme were determined in the preparation phase; the humanistic philosophical movement and the solution-oriented psychological counselling approach were taken as a base in the programme. The draft of the programme implemented in the study was prepared by examining the programmes containing the concepts of problem-solving and emotional resilience, as well as the programmes based on the group studies in the literature, and to finalise the draft, the programme was shaped by means of receiving the opinions of the acknowledged experts in respect of the applicability of the current activities in the programme. In the psychoeducation programme, it is aimed to improve the problem-solving skills and the emotional resilience levels of the participants. The programme content was created in a way that the students in the experimental group can easily understand, and the first two sessions were started with warm-up games so that the students can feel comfortable.

The programme included both *interactive and educational contents*. Activities and practices were developed to reflect a solution-oriented approach. It has been shown that sessions involving children should be carried out using a here-and-now approach, since children are action-oriented and learn by doing and experiencing; fun activities increase their motivation (Friedberg and McClure, 2002). Thus, the study provided imaginative activities, such as drawing. At the end of each session, students provided feedback on the programme by completing a “session evaluation form.” The programme used techniques based on a solution-oriented approach, including exceptional cases, scale, the miracle question, and the cheerleader effect (Table 1).

**TABLE 1:** Programme content.

Sessions	Psychoeducation programme based on the solution-focused approach
1st session	Structuring, objectives of the psychoeducation programme, comprehending solution focused approach
2nd session	Creating purpose, providing information regarding the concepts of emotion and problem
3rd session	Recognising and managing emotions
4th session	Different coping skills and alternative problem-solving skills, exceptional cases
5th session	Predicting the outcomes of certain actions, positive perspective
6th session	Evaluating and concluding the process

The main purpose of the programme was to improve the emotional resilience and problem-solving skills of the fourth- and fifth-grade primary school students by implementing a psychoeducation programme based on a solution-oriented approach. It aimed to enable children to gain knowledge and skills related to recognising and discovering their emotions, controlling their negative emotions, and problem-solving.

## Findings

This section presents research findings on the effect of a psychoeducation programme based on a solution-oriented approach to children’s problem-solving and emotional resilience levels (Table 2).

**TABLE 2:** Descriptive statistics on the problem-solving and emotional resilience pretest-posttest scores of the experimental and control groups.

Groups	Measure	*n*	* $\bar{X}$ *	Ss	Min.	Max.	Skewness	Std. error	Kurtosis	Std. error
Experiment	Problem solving pretest	9	2.96	0.43	2.17	3.46	–0.932	0.717	–0.295	1.4
Experiment	Problem solving posttest	9	3.43	0.39	2.96	4.21	0.764	0.717	0.619	1.4
Experiment	Emotional resilience pretest	9	2.37	0.46	1.68	3.11	–0.201	0.717	–0.553	1.4
Experiment	Emotional resilience posttest	9	2.87	0.69	1.58	4.00	–0.201	0.717	0.736	1.4
Control	Problem solving pretest	9	3.23	0.66	2.25	4.63	1.006	0.717	2.053	1.4
Control	Problem solving posttest	9	3.29	0.94	2.13	4.67	0.572	0.717	–1.134	1.4
Control	Emotional resilience pretest	9	2.72	0.83	1.37	3.95	–0.063	0.717	–0.695	1.4
Control	Emotional resilience posttest	9	2.59	0.84	1.58	3.74	0.160	0.717	–1.48	1.4

The problem-solving pretest score of the experimental group was ($\bar{X}$ = 2.96, Ss = 0.43) and the posttest score was ($\bar{X}$ = 3.43, Ss = 0.39). The emotional resilience pretest score of the experimental group was ($\bar{X}$ = 2.37, Ss = 0.46) and the posttest score was ($\bar{X}$ = 2.87, Ss = 0.69). The problem-solving pretest score of the control group was ($\bar{X}$ = 3.23, Ss = 0.66) and the posttest score was ($\bar{X}$ = 3.29, Ss = 0.94). The emotional resilience pretest score of the control group was ($\bar{X}$ = 2.72, Ss = 0.83) and the posttest score was ($\bar{X}$ = 2.59, Ss = 0.84). According to the results, the experimental group had the highest average score in the problem-solving and emotional resilience posttests (Table 3).

**TABLE 3:** Independent sample *t*-test findings on the examination of the difference in the problem-solving and emotional resilience pretest scores according to the experimental and control groups.

*t*-test	Groups	*n*	* $\bar{X}$ *	sd	*t*	*p*
Problem	Experiment	9	2.96	16	–1.018	0.324
Solving	Control	9	3.23			
Emotional	Experiment	9	2.37	16	–1.101	0.287
Resilience	Control	9	2.72			

The independent samples *t*-test results show no statistically significant difference between the experimental and control groups’ pretest emotional resilience scores (*p* > 0.05); children in the experimental and control groups shared the same characteristics before the psychoeducation programme (Table 4).

**TABLE 4:** Problem-solving and emotional resilience test.

*t*-test	Test	*n*	* $\bar{X}$ *	sd	*t*	*p*
***Experiment***						
Problem	Pretest	9	2.96	8	–2.43	0.041
Solving	Posttest	9	3.43			
***Experiment***						
Emotional	Pretest	9	2.37	8	–2.45	0.040
Resilience	Posttest	9	2.87			

***Control***						
Problem	Pretest	9	3.23	8	–0.342	0.741
Solving	Posttest	9	3.29			
***Control***						
Emotional	Pretest	9	2.72	8	0.683	0.514
Resilience	Posttest	9	2.59			

*The experimental and control group pretest-posttest scores and paired samples t-test findings.*

Unlike the control group, the experimental group showed a positive difference between the pretest and posttest scores (*p* < 0.05) for problem-solving and emotional resilience. This psychoeducation programme, based on a solution-oriented approach, was thus shown to have a positive impact on children’s problem-solving and emotional resilience skills.

## Discussion

This study examined the impact of an online solution-oriented, approach-based psychoeducation programme on the problem-solving and emotional resilience skills of children during the COVID-19 pandemic. As the results show, the online psychoeducation programme had a significant impact on the students’ problem-solving and emotional resilience skills. A review of the literature found that online group work was as effective as face-to-face work (Barak and Wander Schwartz, 1999; Marziali and Donahue, 2006; Greene et al., 2010; Joinson and Paine, 2012). Such results support the findings of this study. Some studies argue that privacy is better protected online (Richards and Vigano, 2013).

Conducting online group work *via* mobile devices has several advantages. It is accessible to individuals as an alternative way of obtaining professional support and offers opportunities to connect with peers, apply acquired skills in the home context, and re-establish social bonds lost during the pandemic period (Wood et al., 2020). In this study, all students in the experimental group perceived themselves as having low levels of social support. These students were able to interact with peers who shared their perceived low levels of social support, interact with friends online during lockdown and quarantine periods, acquire knowledge by absorbing programme content, and transfer their new skills to real life. At the end of the process, the participants gave the programme a positive evaluation. In response to the question: “What was the best part of this session and what did it add to your life?” on the session-evaluation form, one female student mentioned “connecting,” while another referred to “talking and playing”; a male student said, “to socialise.” This feedback is valuable because it shows that students can achieve social bonding even in the online medium. On the objective determination scale, a female student used the following expression to be academically successful: “I need to study, read books and listen to my teachers.” In the 5th session, the students responded as follows. A male student takes turns on the case study form that includes 4 situations: “I’d be upset, I’d look for a solution, I’d get angry and I’d share it with my teacher”; another male student: “I’ll be angry, I’ll warn, I’ll go crazy and the teacher will understand”; and a female student: “please don’t do anything else, and I’ll rewrite it, I’ll tell my mom, I’ll get angry, and I’ll tell the teacher and ask her to postpone it until the next day.” In the event of “Reversing the mind,” a male student used the phrase “I make new friends.”

According to Erzen (2021), online guidance service providers could not observe the bodily reactions of individuals and found it difficult to establish empathy. Although some experts have expressed concerns about the loss of nonverbal information and the lack of intimacy, it is clear that a stronger therapeutic alliance can be established online than in face-to-face studies (Cook and Doyle, 2002; Finn and Barak, 2010; Barak and Grohol, 2011; Wagner et al., 2014). Cui et al. (2010) reported that self-disclosure rates are higher in online groups than in face-to-face consultations. In their meta-analyses, Barak et al. (2008) found that online counselling and face-to-face counselling were equally effective. In this study, the disadvantages of the online psychoeducation programme included the fact that participants’ faces could not be seen clearly, even if the cameras were on; only the upper parts of the students’ bodies could be seen in front of the screen; and some indoor and outdoor sounds could be heard from the students’ homes. However, the participants provided verbal feedback after each session and written feedback after the end of the psychoeducation programme; these show that the process was a positive experience for participants, who left with positive feelings.

Some practitioners are concerned that any disruption to the Internet will have a negative effect on online consultations. For this reason, individuals about to receive online counselling should be told that technology-related problems could occur during the process; precautionary measures should also be explained, along with the advantages and disadvantages of online counselling. This step is important, as it can prevent clients from leaving the process before it is complete (Erdem et al., 2018). In this study, students without computers used their mobile phones to participate in the psychoeducation programme. Before the start of the programme, the researcher explained to members of the experimental group that video communication in technology-supported studies could be interrupted due to Internet-related problems. For this reason, participants without computers needed access to mobile phones, and those using phones needed access to spare phones. In addition, the students were told that it was important to download the psychoeducation programme onto their phones to avoid interruptions. A few students were offline at various times because of faulty Internet connections; they were able to participate in the process when their Internet problems were resolved. WiFi-related Internet problems were resolved using mobile phones. A problem that can occur on the Internet can be considered as a disadvantage of the online process. However, it seems important to consider the fact that online approaches can be as successful as traditional approaches. In this study, no participants dropped out of the process and most said that they would like to be involved in another study.

One limitation of this study was the lack of any action involving the control group. It was impossible to follow up because the study was implemented at the end of the second semester and coincided with the end of the term. A future experimental follow-up study could provide an opportunity to comment on the effectiveness of the implemented programmes. In addition, it can be difficult to create behavioural changes in this age group within a short time. However, behavioural gains could be set as the target in an applied programme, where students would apply taught behaviours in various real-life times and places. The end of the pandemic will allow students to use the skills they have learned in various face-to-face settings. The fact that participants were selected from one school in the designated area, due to the pandemic, is another potential limitation.

At present, the world is changing faster than ever before. On the one hand, technological developments make our lives more convenient; on the other hand, pandemics, wars, forced migrations, and other changes threaten almost every aspect of our social lives. We live in a context of intertwined crises. Long ago, various scientific studies and reports predicted that the globalising world would face more pandemics and that such diseases would affect all societies without discrimination (Budak and Korkmaz, 2020).

Evaluations of the effects of the COVID-19 pandemic on education understand the inevitable impact of postponed school and exam schedules, disrupted teacher training, the burdens of distance education, and social isolation for children. Schools are not simply a place to learn, but a common social area where children learn to socialise; the roles that schools play beyond education are well understood. Studies also acknowledge the role played by various non-traditional teaching methods, such as distance education. The closure of educational institutions has forced many countries to innovate in order to keep their education systems alive. New education systems have begun to take shape all around the world, as countries seek to continue education without interruption.

The pandemic period has shown countries and policymakers that it is possible to maintain education without interruption, even in the face of extraordinary life events, given sufficient investment in distance/online education. Although alternative educational practices are used in times of pandemic, war, and forced migration, they also offer significant advantages in the normal course of life. This study confirms the importance of planning in educational policymaking and practitioner training and the importance of well-trained staff. The findings also show that we must be ready to change what we consider “normal.”

Online psychological counselling is a neglected aspect of many programmes that provide mental healthcare services at TRNC universities. Very few studies have examined the provision of online counselling services in the TRNC. However, existing studies provide valuable evidence on the status of online counselling in the TRNC. When both the advantages and disadvantages are considered, it is clear that online studies complement the traditional approach in extraordinary situations, such as pandemics. To avoid any negative perceptions of studies conducted online or negative experiences among mental healthcare professionals and individuals engaged in online group work, it may be useful to emphasise the importance of conceptual discussions.

The pandemic has shown the countries and policymakers that continuing education without interrupting it in the event of extraordinary life events is possible by investing in online education. Alternative educational practices should not only be considered during the periods of epidemics, wars, and forced migration but also be considered in the normal course of life.

In addition, when the problems of the children and teenagers are examined, there are certain studies analysing the applicability of the psychodynamic approaches (Muratori et al., 2009); however, it must be noted that these approaches will remain limited when implementing them to the kids of primary school age or teenagers. When examining the children of primary school age who are still at the concrete-operational stage in terms of their cognitive levels, their attention span is short, and it is difficult for them to concentrate on something in the long term.

## Author’s Note

This study was produced from the doctoral dissertation conducted in the Guidance and Counseling department of the Institute of Graduate Studies and Research of European University of Lefke.

## Data Availability Statement

Dataset available from the authors upon reasonable request at the permission. Requests to access these datasets should be directed to MÖ, mlhbrk52@hotmail.com.

## Ethics Statement

The studies involving human participants were reviewed and approved by European University of Lefke Institute of Graduate Studies and Research. Written informed consent from the participants’ legal guardian/next of kin was not required to participate in this study in accordance with the national legislation and the institutional requirements.

## Author Contributions

MÖ: ideas and design, analysis of data, interpretation of findings, discussion, conclusion, and reporting of the article. AB: ideas and design, interpretation of findings, discussion, and conclusion. Both authors contributed to the article and approved the submitted version.

## Conflict of Interest

The authors declare that the research was conducted in the absence of any commercial or financial relationships that could be construed as a potential conflict of interest.

## Publisher’s Note

All claims expressed in this article are solely those of the authors and do not necessarily represent those of their affiliated organizations, or those of the publisher, the editors and the reviewers. Any product that may be evaluated in this article, or claim that may be made by its manufacturer, is not guaranteed or endorsed by the publisher.
